# Tumor Cell Heterogeneity in Small Cell Lung Cancer (SCLC): Phenotypical and Functional Differences Associated with Epithelial-Mesenchymal Transition (EMT) and DNA Methylation Changes

**DOI:** 10.1371/journal.pone.0100249

**Published:** 2014-06-24

**Authors:** Alexander Krohn, Theresa Ahrens, Arzu Yalcin, Till Plönes, Julius Wehrle, Sanaz Taromi, Stefan Wollner, Marie Follo, Thomas Brabletz, Sendurai A. Mani, Rainer Claus, Björn Hackanson, Meike Burger

**Affiliations:** 1 Department of Hematology/Oncology and Stem Cell Transplantation, University Medical Center, Freiburg, Germany; 2 Department of Pathology, University Medical Center, Freiburg, Germany; 3 University of Freiburg, Faculty of Biology, Freiburg, Germany; 4 Department of Thoracic Surgery, University Medical Center Witten/Herdecke, Köln, Germany; 5 Department for Visceral Surgery, University Medical Center, Freiburg, Germany; 6 Department of Translational Molecular Pathology, MD Anderson Cancer Center, Houston, Texas, United States of America; 7 University Furtwangen, Faculty of Medical and Life Sciences, Campus VS-Schwenningen, Villingen-Schwenningen, Germany; The University of Arizona, United States of America

## Abstract

Small Cell Lung Cancer (SCLC) is a specific subtype of lung cancer presenting as highly metastatic disease with extremely poor prognosis. Despite responding initially well to chemo- or radiotherapy, SCLC almost invariably relapses and develops resistance to chemotherapy. This is suspected to be related to tumor cell subpopulations with different characteristics resembling stem cells. Epithelial-Mesenchymal Transition (EMT) is known to play a key role in metastatic processes and in developing drug resistance. This is also true for NSCLC, but there is very little information on EMT processes in SCLC so far. SCLC, in contrast to NSCLC cell lines, grow mainly in floating cell clusters and a minor part as adherent cells. We compared these morphologically different subpopulations of SCLC cell lines for EMT and epigenetic features, detecting significant differences in the adherent subpopulations with high levels of mesenchymal markers such as Vimentin and Fibronectin and very low levels of epithelial markers like E-cadherin and Zona Occludens 1. In addition, expression of EMT-related transcription factors such as Snail/Snai1, Slug/Snai2, and Zeb1, DNA methylation patterns of the EMT hallmark genes, functional responses like migration, invasion, matrix metalloproteases secretion, and resistance to chemotherapeutic drug treatment all differed significantly between the sublines. This phenotypic variability might reflect tumor cell heterogeneity and EMT during metastasis *in vivo*, accompanied by the development of refractory disease in relapse. We propose that epigenetic regulation plays a key role during phenotypical and functional changes in tumor cells and might therefore provide new treatment options for SCLC patients.

## Introduction

Small cell lung cancer (SCLC) is a highly malignant and aggressive pulmonary neoplasm accounting for approximately 10–15% of all lung cancers reported. About 30,000 cases are diagnosed per year in the United States [1], [2]. As a separate lung-cancer subgroup distinct from Non Small Cell Lung Cancer (NSCLC, including squamous cell carcinoma, large cell carcinoma, and adenocarcinoma), SCLC is characterized by very early metastasis and poor prognosis. Most patients present with nodal metastases, and two-thirds already have distant metastases at the time of diagnosis [3]. Despite responding initially well to radio- and chemotherapy, the vast majority relapses with a 1-year survival rate for SCLC of only 40%, and 5-year survival under 5% [4]. Whereas NSCLC treatment has progressed in recent years, SCLC treatment remains unsatisfactory [5]. We have witnessed no significant advances in its treatment over the last 30 years since the standard regimen of Etoposide in combination with Cisplatin or Carboplatin was established during the mid-1980s [6]; surgical resection is the exception and might only benefit a minority of careful selected patients [7]. There is obviously an urgent need for new approaches for understanding and treating this specific lung cancer subtype.

SCLC assumes a wide range of morphological appearances histopathologically, with tumor cells usually smaller than the size of 3 resting lymphocytes. However, there are samples containing cells reaching as large as 7 lymphocytes, demonstrating their morphological variability [8] within the tumor tissue. Typical cytological features are finely granulated chromatin, lack of prominent nucleoli and cell borders, and the expression of neuroendocrine markers [8]. Since 2004, SCLC's WHO classification describes two subtypes: pure SCLC with less than 10% large cells, and combined SCLC with over 10% non–small-cell components [9]. Tumors with a high amount of non–small-cell components are described as being more therapy-resistant than pure SCLC [10]. It has also been postulated that SCLC tumors acquire chemo- and radioresistance during treatment, evolving into combined SCLC [11].

To metastasize, cells need to change their phenotype. Individual cells or small groups of cells acquire the ability to migrate and invade through the surrounding tissue's basal membrane. During this process, tumor cells disband cell-cell connections, losing their basal-apical polarity, accompanied by morphologic changes revealing a spindle shape, after which microvilli, filopodia and microtentacles become pronounced instead. Moreover, proteases like Matrix Metalloproteinases (MMPs) become upregulated, facilitating degradation of the extracellular matrix. These processes are also known as the epithelial–mesenchymal transition (EMT) and have been described in a variety of cancer types [12]–[15]. However, there is almost no information on EMT processes in SCLC so far [16]. There is evidence that EMT is critical to both metastasis and in conjunction with chemo- and radioresistance [17]. EMT is a multistage process involving pronounced cellular plasticity and numerous distinct genetic and epigenetic alterations [18]. Transcription factors such as Snail/Snai1, Slug/Snai2, Zeb1, FSP and others cause the up- and downregulation of several genes. E-cadherin has been described as a central factor in the transition between the epithelial and mesenchymal phenotype. Zeb1 and Snail/Snai1 bind to E-cadherin promoter and repress the transcription of cell adhesion molecules [19]. Whereas E-cadherin and Zona Occludens 1 are typically downregulated during EMT, mesenchymal genes such as Vimentin, Fibronectin, and MMPs are upregulated. Epigenetic changes such as DNA methylation, histone modifications and microRNAs are known to be involved in EMT-related gene regulation [15] and therefore might also be responsible for changes in cell phenotype in order to enable spreading and adapting to new micro-environments.

In this study we analyzed different cell subpopulations in SCLC cells lines, especially in NCI-H69 cells, for differences in EMT key genes and functional characteristics. We compared floating-cell aggregates (NCI-H69) with adherently-growing variant subpopulations (NCI-H69V). NCI-H69V was selected from the parental cell line NCI-H69 and is a variant due to low levels of neuroendocrine markers [20]. Comparing the sublines of NCI-H69 clearly reveals a different expression pattern for EMT markers and DNA methylation, which is in turn associated with functional consequences such as the capacity for invasion, migration, MMP secretion and activation, cell proliferation, and chemoresistance. In summary, this study postulates that there is a proportion of mesenchymal cells within SCLC cell lines which might reflect the *in-vivo* situation in SCLC with tumors containing heterogeneous tumor cell subpopulations with more or less epithelial and/or mesenchymal characteristics which might be associated with the functional responses during the metastasis processes.

## Materials and Methods

### Cell culture and reagents

Human SCLC cell lines NCI-H69, NCI-H82 and NCI-N592 were obtained from the American Type Culture Collection (ATCC - Manassas, VA, USA). NCI-H69 and NCI-N592 were verified by LGC Standards Cell Line Authentication. NCI-H69V were kindly provided by the BIOSS Toolbox (Freiburg, Germany). In addition, NCI-H69 and NCI-H69V cells were compared by SNP array (data not shown), which proved same cellular origin. All cell lines were maintained in RPMI (Gibco-BRL, Grand Island, NY, USA) supplemented with 10% Fetal Calf Serum (FCS) and 1% penicillin/streptomycin (Gibco-BRL, Grand Island, NY, USA) in a humidified atmosphere (5% CO2) at 37°C. In order to select for adherent subpopulations, the adherent cells within NCI-H69, NCI-H82 and NCI-N592 were kept in culture, whereas floating cells were disposed over multiple passages.

### Growth rates and population doubling time

Growth rates of NCI-H69 and NCI-H69V were determined seeding 3*10^6^ cells (in triplicates) into tissue culture flasks. Cells were counted on day 2, 4 and 6 using a hemocytometer. To calculate population doubling time (PDT), the formula PDT = h*ln(2)/ln(c2/c1) was used according to ATCC guidelines.

### Immunofluorescence staining of Vimentin, E-cadherin, Zona Occludens and Ki-67

Cells were fixed in 2% PFA, permeabilized with 0.5% TritonX-100 at 4° for 10 min, and blocked with PBS containing 5.0% (v/v) normal goat serum and 0.3% (v/v) Triton X-100. Immunofluorescence staining was performed with mouse mAb Ki-67 (Dako, Hamburg, Germany), Vimentin XP Rabbit mAb, E-cadherin rabbit mAb and Zona Occludens mAb (Cell Signaling Technologies, MA, USA), and appropriate secondary antibodies (goat-anti-mouse IgG-Alexa488 and goat-anti-rabbit IgG-Alexa467, Invitrogen, Karlsruhe, Germany). The slides were then mounted with Prolong Gold Antifade Reagent with DAPI (Invitrogen Life Technologies, Carlsbad, CA, USA).

### Reverse transcription PCR analysis

Total RNA was isolated using the RNeasy Mini Kit (Qiagen, Hilden, Germany) according to the manufacturer's instructions, followed by DNA digestion with DNase I (Applied Biosystems, Warrington, Cheshire, UK). For cDNA synthesis 1 µg of total RNA was transcribed using the iScript kit (*Bio-Rad*, Hercules, CA, USA). The cDNA was amplified using *Taq* polymerase (Qiagen, Hilden, Germany) using following PCR program for all primers: 94°C for 5 min followed by 28 cycles of 94°C 30 sec, 55°C 30 sec and 72°C for 30 sec and last round of amplification at 72°C for 5 min. PCR products were analyzed on a 1.0% agarose gel, visualized, and photographed under UV light. For quantification, PCR bands were analyzed by ImageJ 1.42q. For primer sequences see Table 1.

**Table 1:** Primers used for RT-PCR.

	Primers (5′-3′)	
	Sense	Antisense
Vimentin	GAGAACTTTGCCGTTGAAGC	GCTTCCTGTAGGTGGCAATC
Fibronectin	CAGTGGGAGACCTCGAGAAG	TCCCTCGGAACATCAGAAAC
FSP-1	GGTGTCCACCTTCCACAAGT	GCTGTCCAAGTTGCTCATCA
Snail/Snai1	GCG AGC TGCAGGACTCTAAT	GGACAGAGTCCCAGATGAGC
Slug/Snai2	GGTCAAGAAGCATTTCAAC	GGTAATGTGTGGGTCCGA
E-cadherin	AGGCCAAGCAGCAGTACATT	ATTCACATCCAGCACATCCA
MMP-1	CTGAAGGTGATGAAGCAGCC	AGTCCAAGAGAATGGCCGAG
MMP-2	GTGCTGAAGGACACACTAAAGAAGA	TTGCCATCCTTCTCAAAGTTGTAGC
MMP-9	GACGCAGACATCGTCATCCA	AACTCGTCATCGTCGAAATGG
MMP-10	GTCCTTCGATGCCATCAGCA	CTTGCTCCATGGACTGGCTA
MMP-13	ATGCATCCAGGGGTCCTGGC	TGCTCGCATTCTCCTTCAGGA
MMP-14	GAGAGGAAGGATGGCAAATTC	CAATGATGATCACCTCCGTCT
GAPDH	ACCCAGAAGACTGTGGATGG	TCTAGACGGCAGGTCAGGTC

### Preparation of cell extracts and western blot analysis

Cells were washed with ice-cold PBS, and lysed using Qproteome Mammalian Protein Prep Kit (Qiagen, Hilden, Germany) or by lysis buffer containing 20 mM Tris/HCl pH 8.0, 150 mM KCl, 1 mM EDTA, 0.2 mM Na3VO4, 1% Triton X-100, 0.5 mM PMSF with protein inhibitor cocktail (complete, Roche Applied Science, Basel, Switzerland). Equal amounts of protein samples were denatured at 95°C for 5 minutes, separated by 10% SDS-PAGE and transferred onto PDVF membranes. Membranes were then blocked with 5% non-fat dry milk in PBS/0.1% Tween-20 and incubated overnight with the following antibodies: ZO-1 mAB, Vimentin XP rabbit mAb, E-cadherin rabbit mAb, Snail rabbit mAb, Slug rabbit mAb, TCF8/ZEB1 Rabbit mAb, β-Catenin rabbit mAb Anti-rabbit IgG, GAPDH rabbit mAb, HRP-linked Antibody (Epithelial-Mesenchymal Transition (EMT) Antibody Sampler Kit #9782 from Cell Signaling Technologies, MA, USA) and L-Dopa (#8786 Cell Signaling Technologies, MA, USA), Neuron-specific enolase (NSE) (#9536 Cell Signaling Technologies, MA, USA). Finally, immunoreactive bands were visualized using horseradish peroxidase-conjugated secondary antibodies and the enhanced chemiluminescence system (Amersham Biosciences, Freiburg, Germany).

### Pyrosequencing

Genomic DNA was extracted using DNeasy Blood & Tissue Kit (Qiagen, Hilden, Germany) and quantified using a NanoDrop 1000 Spectrophotometer (peqlab, Erlangen, Germany). DNA was bisulfite-treated using the EZ DNA Methylation-Gold Kit (Zymo Research, CA, USA) according to the manufacturer's instructions and stored in aliquots at −20°C until use. PCR Primers were designed using Pyrosequencing Assay Design Software (Qiagen, Hilden,Germany) (Tab.2). A universal tag was placed at the 5′end of the sequence-specific reverse primer for CDH1 and a corresponding universal biotinylated primer containing a 5′ biotin label was added to these PCR reactions. The 5′end of the sequence-specific reverse primer used for VIM amplicon 1 and amplicon 2 was biotin labeled. The amplicon (spanning −61 to +18 with respect to the TSS) of CDH1 included seven CpG sites. In case of Vimentin, the amplicon was divided into two parts, separated by a 73-bp gap. The first amplicon of Vimentin between nt +608 to +703 with respect to the TSS contained twelve and the second amplicon (spanning +468 to +537 with respect to the TSS) eight CpG sites, respectively. Each 25 µl PCR reaction contained 1.5 mM MgCl2, 0.4 mM dNTP, 0.03 U/µl Hot start Taq DNA polymerase (Invitrogen, CA, USA), 0.16 µM forward and reverse primer and 1 µl of bisulfite treated DNA. For CDH1 0.16 µM forward primer and 0.03 µl tailed reverse primer and 0.14 µM universal biotinylated primer was used. Cycling conditions were as follows: 95°C for 5 min for initial denaturation; 50 cycles of denaturation at 94°C for 30 seconds, varying annealing temperature for 30 s and extension at 72°C for 30 s. PCR products were visualized on 2% agarose gels. Every pyrosequencing reaction was obtained with 8–10 µl of each biotinylated PCR product and the Pyromark Gold Kit according to the manufacturer's instructions on a PyroMark Q96 MD pyrosequencer (Qiagen, Hilden, Germany). In brief, biotinylated single strand DNA was immobilized on streptavidin-coated Sepharose High Performance beads (GE healthcare, UK) and separated by denaturation with 0.2 N NaOH. After annealing of the sequencing primer (0,25 µM/reaction) to the biotinylated single-strand DNA, the mix was subjected to sequencing. Pyrograms were analyzed using the Pyro Q-CpG software to determine the methylation level of methylated and unmethylated cytosines at each CpG site in the amplicon. Each pyrosequencing analysis was independently performed at least three times. Student's unpaired t tests were conducted to test statistical differences in the mean methylation level of the analyzed amplicons. Cultured lung fibroblasts isolated from three patients without fibrotic diseases served as normal control. The primer pairs used for PCR are listed in Table 2.

**Table 2:** Primers used for pyrosequencing.

			Tm [°C]	amplicon [hg19]
CDH1	forward primer(5′- 3′)	GGTTGTGGTAGGTAGGTGAATT	56°C	chr16:68,771,134–68,771,212
	reverse primer (5′-3′)	GTGCCAGGCTCAGGCAAACTAACTTCCCCAAACTCACAAATACTT	56°C	
	universal biotinylated primer (5′-3′)	BTN-ATCTGTGCCAGGCTCAGGC	56°C	
	sequencing primer (5′- 3′)	GGTAGGTGAATTTTTAGTTAA		
VIM amp1	forward primer(5′- 3′)	GGGTTTATTTTGGGGTGTTGAA	60°C	chr10:17,270,868–17,270,957
	reverse primer (5′-3′)	BTN-ACTTCAAATCTAAAAAATTCCTTACTCT	60°C	
	sequencing primer (5′- 3′)	GTTTGGGATTGAATTAGAG		
VIM amp2	forward primer(5′- 3′)	GGGAAGAGGAAAGAGTAAGGAATTT	56°C	chr10:17,270,725–17,270,794
	reverse primer (5′-3′)	BTN-ACAACCCCCCTTTCCAAAC	56°C	
	sequencing primer (5′- 3′)	AATAAAGAGAGTTTGAGATTGG		

### Live-cell proteolysis assay

LabTek 4-well chamber slides were coated with phenol-red free basement membrane matrix, containing 30 ng/ml DQ-Collagen IV (Invitrogen Life Technologies, Carlsbad, CA, USA). After 12 min solidification, 3×10^4^ cells in phenol-red free RPMI with 1% FCS were plated on top of the coated basement membrane matrix. After 48 h incubation, cells were fixed with 2% PFA, stained with Hoechst and mounted. Degradation products of the DQ-substrate (green fluorescence) were analyzed on an automated microscope (scan∧R - Olympus) using a 20x, N.A. 0.75 UPLSAPO objective with Hoechst emission measured at 440–475 nm and DQ-Collagen at 520–550 nm. The mean intensity of DQ-substrate degradation was calculated using the scan∧R Analysis Software (v. 1.2.0.6) and normalized to the number of cell nuclei.

### Matrix metalloproteinase antibody array

Matrix metalloproteinases in cell-conditioned media were analyzed with the Human MMP Array 1 (RayBiotech, Norcross, GA, USA). Cells were washed with PBS and incubated with FCS-free and phenol-red free RPMI 1640 overnight. After centrifugation, cell conditioned medium was applied to the pre-blocked membranes, incubated at 4°C overnight and processed according to the manufacturer's instructions. Antibody arrays were quantified with ImageJ software v. 1.42q.

### Gelatine Zymography

MMP-2 and MMP-9 activities were assessed by gelatin zymography. Cells were washed with PBS and incubated with FCS-free and phenol-red free RPMI 1640 overnight. Cell conditioned media were concentrated using Amicon Ultracel 10 k columns. After determining protein concentrations by Bradford assay, equal amounts of protein and 2 µl MMP-2 Zymography Control (ProteaImmun, Berlin, Germany) were electrophoresed in 7% SDS-polyacrylamide gels, containing 1 mg/ml gelatin (Merck, Darmstadt, Germany). Gels were washed in 2.5% Triton X-100 solution for 1 hour, then incubated overnight in incubation buffer (0,05 M Tris/HCl pH 7,5, 0,2 M NaCl and 0,005 M CaCl2) at 37 °C. Gels were fixed in 12% TCA for 1 hour and stained in Coomassie brilliant blue-R250 staining solution for 1 hour, followed by destaining. For loading controls, samples were loaded onto SDS-PAGE and stained by Coomassie brilliant blue-R250 staining.

### Drug Resistance

To test chemosensitivity, 3×10^4^ NCI-H69 or NCI-H69V cells were seeded in 6 well plates for 12 h and were then treated with 200 µM Etoposide (Sigma-Aldrich, Munich, Germany) for 48 h as described before [21]. Afterwards, cell viability was tested using propidium iodide (PI) (Sigma-Aldrich, Munich, Germany) and 3,3′-dihexyloxacarbocyanine iodide (DIOC6) (Sigma-Aldrich, Munich, Germany). Cells were incubated in RPMI 1640 containing 0.5% BSA, 10 nM DIOC 6 and 2 mg/ml PI at 37°C for 20 min and analyzed by flow cytometry on a FACSCalibur (Becton Dickinson, Mountain View, CA, USA). Flow cytometry data were analyzed using FlowJo software v.7.6.3 (Tree Star Inc., San Carlos, CA, USA).

### Transwell migration

Migration of SCLC cells was evaluated using 24-well microchemotaxis plates (Costar, New York, USA), in which 1×10^6^ cells in 100 µL RPMI 1640 without FCS after starvation for 12 hours were put into the upper chamber and the lower chamber contained RPMI with 10% FCS. After 24 h of incubation at 37°C in 5% CO_2_, cells were counted according to the manufacturer's instructions. Invaded cells at the bottom of the insert membrane were dissociated with trypsin and counted using a hemocytometer. Migration Index was calculated as the number of migrated cells divided by number of migrated cells in the untreated group. Each experiment was done in triplicate. Data represent mean +/− SD from three independent experiments.

### Invasion assays

For invasion assays the QCMTM 24-Well Cell Invasion Assay (ECM 550; Chemicon International, Temecula, CA, USA) with an 8 µM pore size polycarbonate membrane and a layer of ECMatrix-like material was used. Assays were done according to the manufacturer's instructions, by which 6×10^5^ cells were starved for 12 h, seeded into the upper chamber, and incubated for 48 h at 37°C and 5% CO_2_. The lower chamber contained RPMI with 10% FCS. After 48 h cells in the upper chamber were removed using cotton swabs, invaded cells at the bottom of the insert membrane were dissociated with trypsin and counted using a hemocytometer. Experiments were performed in triplicate.

### Statistical analysis

Statistical significance between controls and individual conditions was assessed by GraphPad Prism 5 Software using the t-test. * represents a P value from 0.01 to 0.05, ** represents a P value from 0.01 to 0.001 and *** represents a P value less than 0.001. A p value of <0.05 was considered to be statistic significant.

## Results

### The key EMT markers Vimentin and E-cadherin are differentially expressed

**Figure 1:**
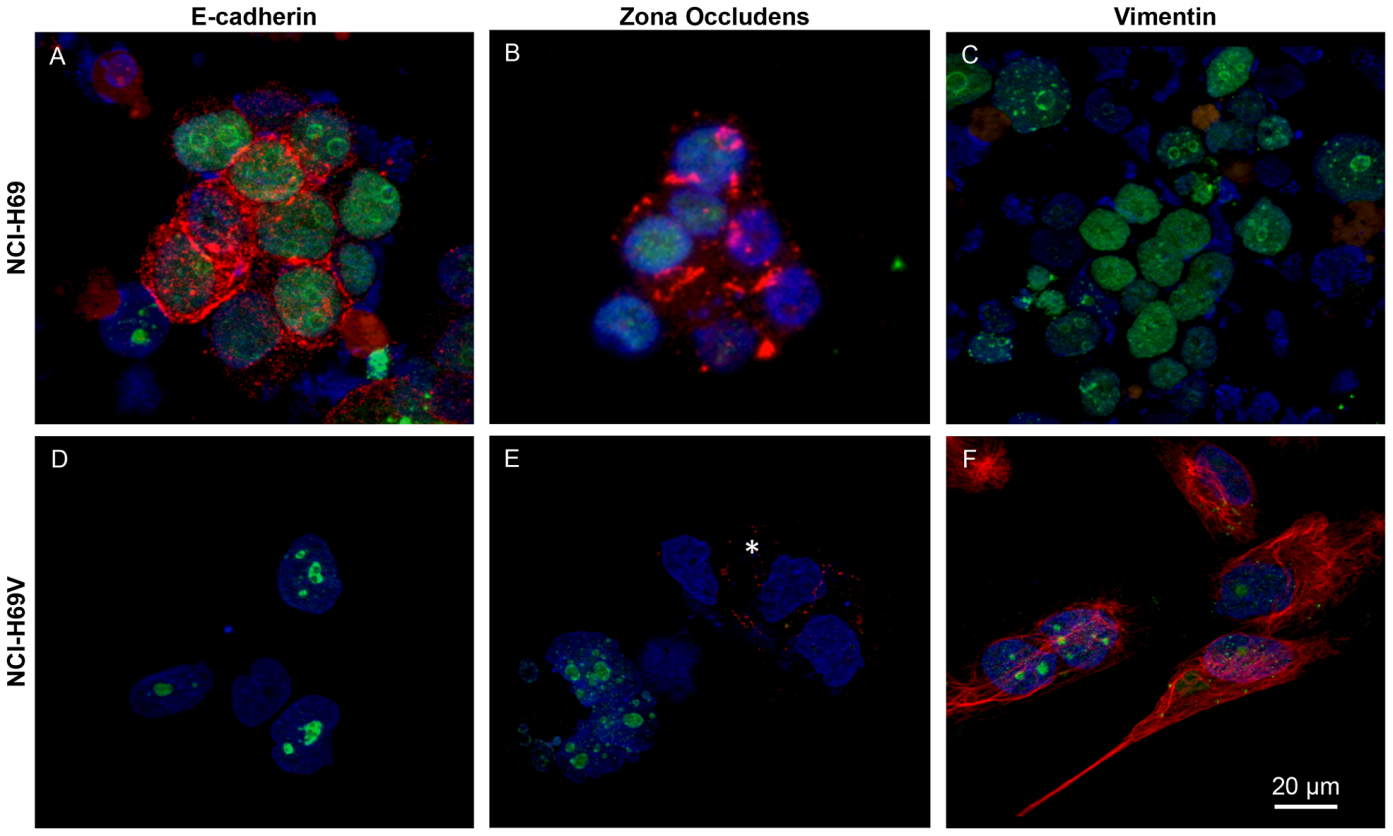
Immunofluorescence staining for EMT markers. Suspension cells (NCI-H69) are strongly positive for E-cadherin (red) (**a**) and Zona Occludens (red) (**b**), whereas adherent NCI-H69V cells are negative for E-cadherin (**d**). Majority of NCI-H69V cells were negative for Zona Occludens, but some single cells showed slight Zona Occludens expression (asterisk, **e**). Suspension cells (NCI-H69) are negative (**c**), whereas adherent NCI-H69V are positive (**f**) for Vimentin (red). Ki-67 was co-stained (green). Cells were imaged by Zeiss Axioplan microscope and AxioCam Digital and analyzed by ZeissAxioVision software. Bar represents 20 µm.

SCLC cell lines such as NCI-H69 typically grow as tightly-packed floating aggregates. However, a consistent proportion (5–10%) of the total observable cell number grows adherently. For the cell line NCI-H69 an adherent subline has been established named NCI-H69V [20]. Immunofluorescent staining differed considerably when comparing NCI-H69 and its subline NCI-H69V for the key markers of epithelial and mesenchymal cell phenotypes E-cadherin and Vimentin: NCI-H69 were positive for E-cadherin (Figure 1a) and Zona Occludens (Figure 1b) but showed no Vimentin expression (Figure 1c), whereas the adherent NCI-H69V was negative for E-cadherin (Figure 1d) but strongly positive for Vimentin (Figure 1f). Majority of NCI-H69V cells were negative for Zona Occludens (Figure 1e); however some single cells showed slight remaining Zona Occludens expression (Figure 1e, asterisk). Expression of E-cadherin and Vimentin, as hallmark genes of EMT, distinguishes between the different subpopulations within the cell line, demonstrating a majority of epithelial-like cells and fewer adherently-growing cells with a mesenchymal-like phenotype.

### Different cell morphology within SCLC cell lines

**Figure 2:**
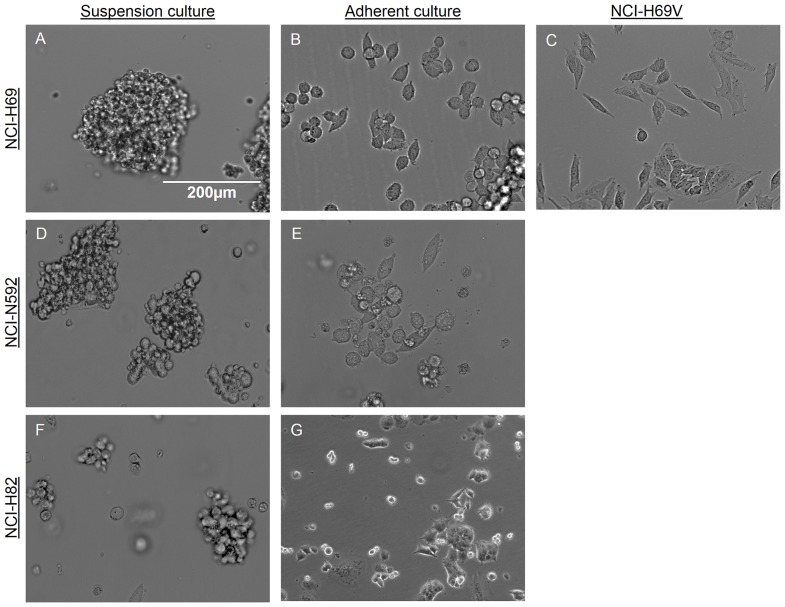
Morphologic differences within SCLC cell lines. NCI-H69 SCLC cell lines are growing in floating clusters (**a**) with a small subpopulation of adherently-growing cells that were selected by disposing floating cells over multiple passages (**b**) resembling the established subline NCI-H69V (**c**). NCI-N592 and NCI-H82 in typical floating clusters (**d, f**) and selected adherent cells (**e, g**). Adherent subpopulations reveal a spindle shape, filopodia and microtentacles. Cells were imaged by Zeiss Axioplan microscope and AxioCam Digital and analyzed by Zeiss LSM Image Browser. Bar represents 200 µm.

We investigated the cell morphology of subpopulations (floating vs. adherent) in SCLC cell lines. For this purpose the adherent subpopulation was increased by selection until the cells grew as a completely adherent monolayer (Figures 2b, e, g). The cell lines showed differences in spontaneous adherence: NCI-N592 attached more frequently and quicker than NCI-H69 (NCI-H69: Figures 2a, b/NCI-N592: Figures 2d, e). An adherent subline has been established for NCI-H69, namely NCI-H69V (Figure 2c) [20]. The adherent cell line we selected ourselves revealed similar morphology (Figures 2b, c). Growth rates and doubling times of NCI-H69 and NCI-H69V were comparable with 28 h (±6,02) for NCI-H69 and 30 h (±7,34) for NCI-H69V (Figure S1).

There are impressive morphological differences between adherent and floating cells in all investigated SCLC cell lines: whereas cells in floating clusters appear small and round, with small nuclei, attached cells are larger, spread out, and have a large cytoplasm-to-nucleus ratio. In particular, NCI-H69V cells have a spindle shape with long pseudopodia and fibers, fine granular chromatin and a lack of prominent nucleoli. In addition, adherent cells do not grow via tight cell-cell contacts until they are highly confluent.

### Expression of EMT-related genes and DNA methylation changes in Vimentin and E-cadherin during EMT

**Figure 3:**
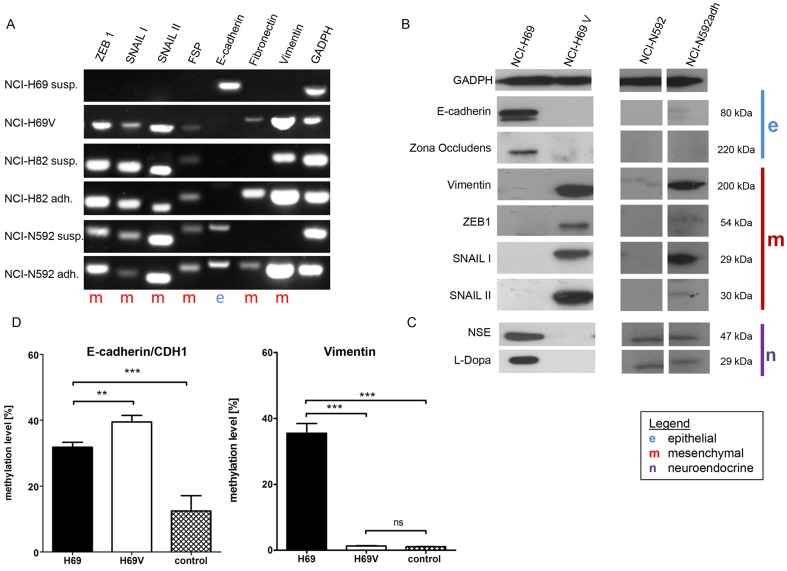
Expression and methylation of EMT and neuroendocrine markers in different SCLC cell lines. EMT markers and transcription factors are expressed differently within the SCLC lines (NCI-H69, NCI-H82, and NCI-N592). NCI-H69 cells did not express mesenchymal markers or inductors in suspension cultures, evident on the mRNA level by RT-PCR (**a**). Zeb1, Snail/Snai1, Slug/Snai2, and FSP are expressed in the adherent subline (NCI-H69V), but E-cadherin is only expressed in suspension cells. Mesenchymal markers Fibronectin and Vimentin are upregulated in the adherent sublines (**a**). The mRNA expression in NCI-H82 and NCI-N592 compared with its adherent subcultures generally revealed a similar tendency toward a mesenchymal phenotype in the adherent cells (**a**). Western blot analysis displays decreased protein levels in the epithelial markers E-cadherin and Zona occludens 1, and expression of mesenchymal markers Vimentin and ZEB1 in NCI-H69V. EMT inductors Snail/Snai1, Slug/Snai2 are expressed in NCI-H69V (**b**). The expression change in adherent NCI-N592 toward a mesenchymal-like phenotype is more significant on the protein level than the mRNA level (**b**). The neuroendocrine markers NSE and L-Dopa are expressed in the parental cell line NCI-H69, but are not detectable in the adherent subline NCI-H69V (**c**). DNA methylation analysis (**d**) of the EMT hallmark gene Vimentin demonstrates strong methylation in NCI-H69 and significant hypomethylation in NCI-H69V, E-cadherin methylation is significantly higher in H69V. As control, cultured lung fibroblasts isolated from three patients for Vimentin methylation levels and from two patients for E-cadherin methylation levels without fibrotic diseases served as normal control. The bar shows the mean of three independent measurements. The differences between the mean methylation levels of the analyzed amplicon was tested using Student's unpaired t-test (* p value from 0.01 to 0.05, ** p value from 0.01 to 0.001 and *** p value less than 0.001).

mRNA expression of typical EMT gene products were compared between the different SCLC cell lines and its selected adherent sublines by RT-PCR (Figure 3a). The epithelial marker gene E-cadherin was highly expressed in floating NCI-H69. In contrast, E-cadherin expression was low in adherent NCI-H69V. Vimentin was absent in the floating NCI-H69 cells, whereas it was strongly expressed in adherently growing NCI-H69adh and NCI-H69V (Figure 3a). These differences in mRNA expression were also detected in NCI-H82 and NCI-N592 sublines, with the adherent cells demonstrating a more mesenchymal-like pattern. However, the conversion was not as clear as that in NCI-H69/NCI-H69V (Figure 3a).

Taken together, the EMT-related transcription factors Zeb1, Snail/Snai1, Slug/Snai2 and FSP were expressed variably in the different cell lines (Figure 3a). We noted the strongest differences in NCI-H69V in comparison to NCI-H69 and therefore focused on those cells in the following experiments.

This conversion from epithelial NCI-H69 cells into a mesenchymal phenotype (NCI-H69V) was demonstrated even more obviously on the protein level (Figure 3b): we detected significant downregulation of the epithelial markers E-cadherin and Zona occludens 1 in NCI-H69V accompanied by upregulation of EMT inductors and effectors such as ZEB1, Snail/Snai1, Slug/Snai2 and Vimentin. Although the mRNA expression of NCI-N592 only tended toward a mesenchymal phenotype (Figure 3a), protein expression clearly revealed a mesenchymal pattern (Figure 3b).

Interestingly, the neuroendocrine markers NSE and L-Dopa were expressed in the parental cell line NCI-H69, but were not detectable in its adherent subline (Figure 3c).

### DNA methylation of Vimentin and E-cadherin

To assess whether epigenetic regulation by DNA promoter methylation might play a role in expression levels, we applied quantitative pyrosequencing to the promoter regions of Vimentin and E-cadherin (Figure 3d). While the Vimentin promoter showed DNA methylation levels of 35% in NCI-H69 cells, DNA methylation levels in NCI-H69V cells were significantly lower with 1,3% (p<0.0001). This leads to active gene transcription, which falls in line with the observed increase in Vimentin on the mRNA/protein level (Figure 1 and 2). Normal lung fibroblasts (n = 3) showed Vimentin methylation levels of 1.1%. Conversely, DNA methylation levels of E-cadherin/CDH1 were 31% in H69 cells and increased to 40% in NCI-H69V cells (p = 0.0032). Normal lung fibroblasts (n = 2) showed 17% and 8% methylation, respectively. This inverse correlation between expression and DNA methylation may suggest that DNA methylation contributes to Vimentin and E-cadherin mRNA regulation and protein expression during the EMT process in SCLC cell lines.

### Higher migration and invasive potential of adherent NCI-H69

**Figure 4:**
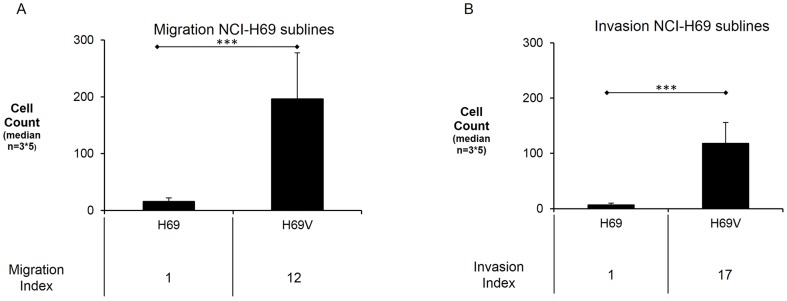
Increased migration and invasion in adherent NCI-H69V sublines. Adherently-growing NCI-H69 cells demonstrate significantly higher migration (**a**) and invasion (**b**) towards 10% FCS containing medium compared to the suspension cell line (*** p = .0001). Migration Index was defined as number of migrated cells divided by number of migrated cells in the untreated group (no FCS) after 48 hours. Invasion Index was defined as number of counted cells in five view fields divided by the number of migrated cells in the untreated group (no FCS) after 48 hours.

To investigate whether these dramatic phenotypic differences have functional consequences, we carried out transwell migration and invasion assays. The adherent NCI-H69V cells demonstrated significantly higher migration and invasion into extracellular matrix proteins towards 10% FCS-containing medium than the NCI-H69 cells (Figure 4). NCI-H69 showed very little migration potential and nearly no invasion. The migration level in NCI-H69 suspension cells was 15 cells per migration chamber (±6/n = 3), whereas NCI-H69V rose 11-fold with 196 cells per migration chamber (±81/n = 3) (p = 0.0001). The invasion level in H69 was 7 cells per migration chamber (±3/n = 3), whereas NCI-H69V was 16-fold higher with 118 cells per migration chamber (±35/n = 3) (p = 0.0001).

### Live cell imaging of extracellular proteolytic activity indicates higher proteolytic potential of adherent sublines

**Figure 5:**
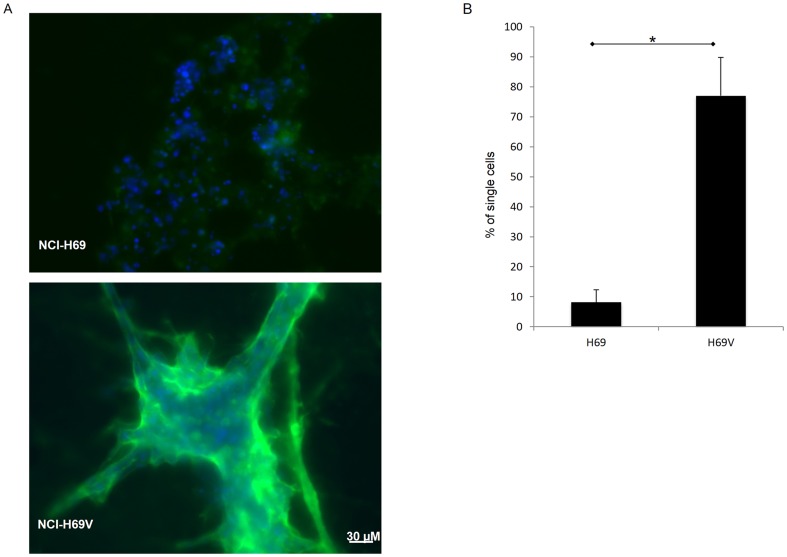
Extracellular proteolytic activity comparing NCI-H69 and NCI-H69V sublines. Live-cell proteolysis assay: suspension NCI-H69 and adherent NCI-H69V cells were incubated for 48 hours on matrigel containing 30 ng/µl DQ-collagen IV. (**a**) Degradation of collagen is determined by green fluorescence that surrounds positive cells. Bar represents 30 µm. (**b**) Diagram shows mean values of three independent experiments with standard deviation.

Cancer cell invasion is accompanied by degradation of Extracellular Matrix (ECM). Because of greater invasion of the adherent subline, we analyzed its potential for collagen IV degradation. After 48 h of incubation on matrigel containing DQ-collagen IV, adherent NCI-H69V cells revealed significantly higher pericellular degradation of collagen than NCI-H69 cells as measured by green fluorescence. Quantification of proteolytically active cells revealed that 77% of the NCI-H69V cells were positive, whereas only 8% of the NCI-H69 suspension cells were positive (Figure 5).

### Adherent sublines show increased expression and secretion of MMPs

**Figure 6:**
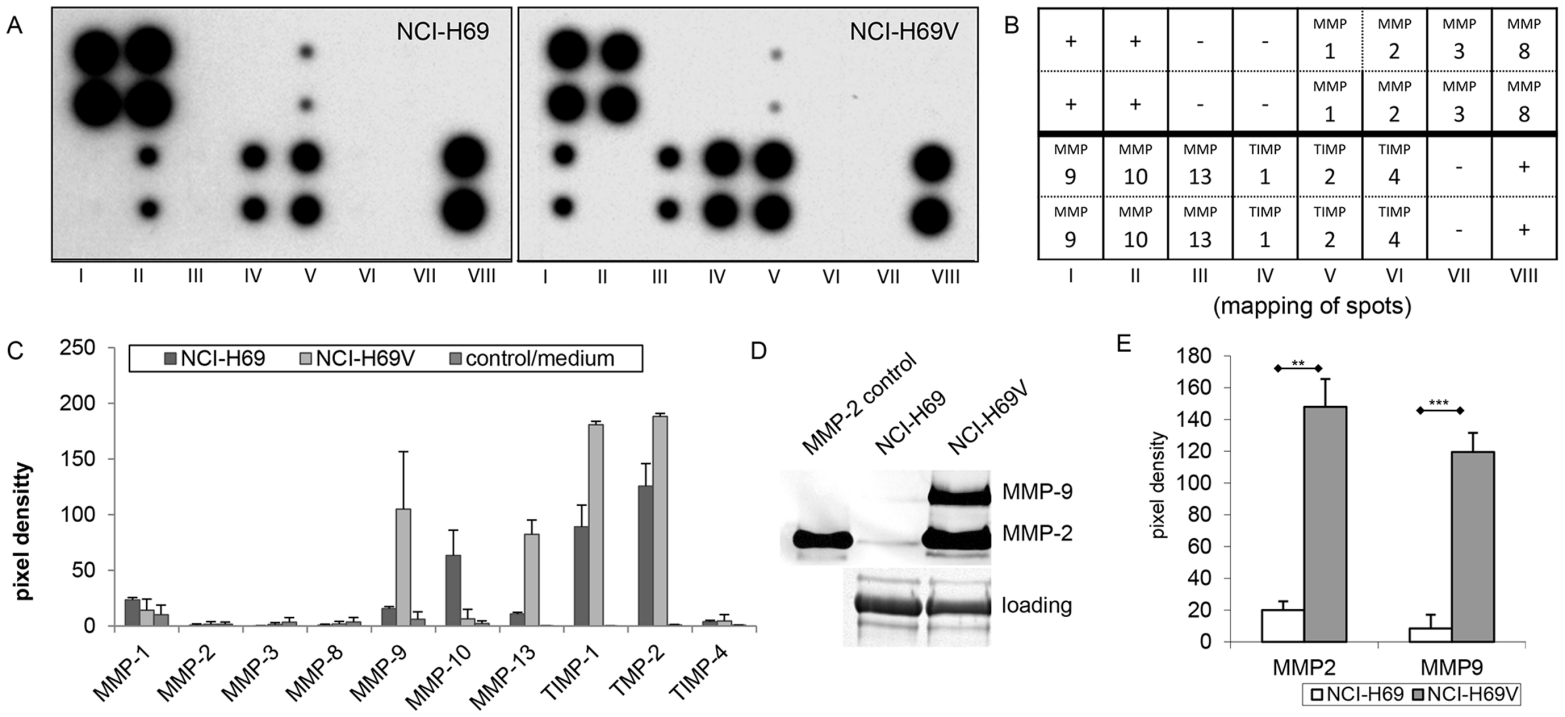
Secretion and activation of MMPs by NCI-H69 and NCI-H69V sublines. Secreted MMPs were analyzed in NCI-H69 suspension and NCI-H69V cells by antibody arrays (**a**). Mapping of spots (**b**), antibody arrays were then quantified by ImageJ. The bars represent means of two independent experiments (**c**), white bar: NCI-H69/gray bar: NCI-H69V/black bar: medium control. Activation of MMP-2 and MMP-9 were determined by gelatin zymography (**d**) and quantified by ImageJ (**e**). The diagram shows mean values of three independent experiments with standard deviation (**e**).

MMPs are known to be associated with increased invasion and metastasis in different cancer entities. To evaluate whether MMPs are involved in SCLC invasion and increased proteolytic activity, we analyzed MMP expression and secretion. Analysis of mRNA expression by RT-PCR revealed strong upregulation of MMP-1, MMP-2, MMP-9, MMP-13 and MMP-14 in the adherent NCI-H69V cells and of MMP-1, MMP-2 and MMP-13 in adherent NCI-N592 cells (Figure S2a). Moreover, secreted MMPs were determined in cell-conditioned medium (CCM) by MMP antibody arrays (Figure 6a). Consistent with the observed mRNA expression levels, MMP-1, MMP-9 and MMP-13 were more abundant in the CCM of NCI-H69V cells (Figure 6c). Furthermore, the amounts of tissue inhibitors in metalloproteinases (TIMP-1, TIMP-2 and TIMP-4) were elevated (Figure 6c). For MMP-2, we did a control western blot that revealed MMP-2 being more secreted into the CCM in NCI-H69V cells (Figure S2b). In addition, gelatin zymography confirmed MMP-2 and MMP-9 activation. Indeed, the bands characteristic for active MMP-2 and MMP-9 were significantly enhanced in adherent NCI-H69V compared to floating NCI-H69 cells (Figure 6d and e). Membrane-bound MMP-14 is required to activate MMP-2 and is known to be involved in cancer invasion. We observed a 2.2-fold change in MMP-14 mRNA levels and a 13.8-fold change in MMP-14 protein levels in NCI-H69V compared to NCI-H69 suspension cells (representative PCRs and western blots are shown in Figure S2a,b). In summary, the adherent NCI-H69V subline demonstrated increased expression and secretion of MMPs compared to the parental NCI-H69.

Taken together, the migration, invasion, degradation of ECM and MMP expression are in line with observed EMT marker changes towards a mesenchymal-like subpopulation, represented by NCI-H69V cells.

### Increased drug resistance of adherent NCI-H69V cells

**Figure 7:**
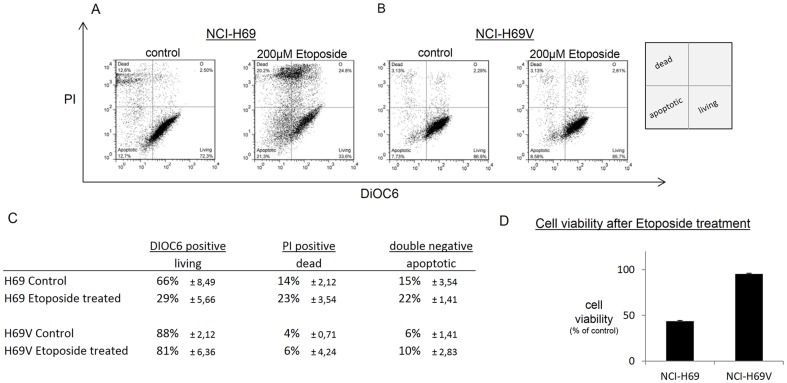
Higher chemoresistance of H69V cells compared to NCI-H69. NCI-H69V treated with 200 µM Etoposide showed significantly increased chemoresistance in comparison to NCI-H69 cells. After 48 h treatment with Etoposide, cells were stained with Propidium iodide and DiOC6 to detect vital, dead and apoptotic cells via flow cytometry. Dot plots of one representative experiment repeated three times (**a, b**). Mean values from the three independent experiments of vital, dead and apoptotic cells are displayed as percentage of untreated controls (**c**). Cell viability of NCI-H69 and NCI-H69V after Etoposide treatment expressed as mean values and standard error of three independent experiments (**d**).

Another feature many tumors acquire during EMT is drug resistance. We analyzed resistance to Etoposide, a standard chemotherapeutic drug in SCLC therapy. NCI-H69 and NCI-H69V cells were treated with 200 µM Etoposide for 48 h before testing cell viability via DIOC6/PI staining (Figure 7a: NCI-H69, 7b: NCI-H69V). Cell viability fell to 29% (±5,66%) after 48 h treatment compared to 66% (±8,49%) in the untreated NCI-H69 suspension. Cell viability in the adherent NCI-H69V remained at 81% (±6,36%) compared to 88% (±2%) in untreated cells after 48 h treatment (Figures 7c–d), indicating that the adherent subline is more drug-resistant than NCI-H69 suspension cells (Figure 7), thus proving drug resistance as one characteristic of EMT [22].

## Discussion

Primary tumors and metastases are often composed of heterogeneous cell populations containing differentiated and dedifferentiated cells, particularly located at the invasive front. The latter cells are described as displaying those hallmarks of EMT associated with increased tumor-cell dissemination [19].

SCLC cell lines in culture always reveal cell heterogeneity, with a majority of cells growing in floating cell aggregates (Figures 2a,d,f) and a smaller fraction of adherently growing cells (Figures 2b,c,e,g). The percentage of adherent cells depends on factors such as hypoxia, malnutrition, cell density and other stress factors [20].

In our study we examined the composition of SCLC cell lines and their adherent sublines with regard to EMT markers. As the cell lines differed significantly in expressing epithelial markers such as E-cadherin and Zona Occludens and mesenchymal markers like Vimentin (Figure 1), we hypothesized that these sublines might display tumor-cell heterogeneity occurring *in vivo*. We are aware that this is an artificial *in vitro* cell line model and *in vivo* heterogeneity is never restricted to these two extremes with clear cut epithelial and mesenchymal characteristics. Nevertheless, these cell sublines might be a useful tool to study phenotypical and functional differences.

There is evidence that EMT processes, including the ability of cells to invade and migrate into surrounding tissues, are accompanied by changes in cell morphology [13], [14], yet very little is known about EMT processes in SCLC. Nevertheless, it could be essential to understanding the unique clinical behavior of SCLC compared to other lung cancer entities. The spindle-shaped appearance, formation of microtentacles and loss of basal-apical polarity were observed in the adherent SCLC cell sublines (NCI-H69V, and the adherent sublines of NCI-H69, NCI-N592, and NCI-H82) with mesenchymal characteristics (Figure 1, 2). In contrast, the floating cell aggregates in the cell lines (NCI-H69, NCI-N592) showed characteristics of epithelial-like cells (Figures 1, 2).

Further analyses of EMT-marker expression at mRNA and protein levels in the adherent sublines revealed that typical epithelial markers like Zona occludens 1 and E-cadherin were downregulated, whereas Vimentin as a typical mesenchymal marker was upregulated (Figures 3a, b). In contrast, the floating and tightly-packed cells demonstrated high E-cadherin and Zona occludens 1 expression. Changes from E-cadherin expression towards Vimentin expression is a hallmark characteristic of EMT [23], [24]. The transmembrane glycoprotein E-cadherin is characteristic of epithelial cells. It functions in maintaining regular epithelial tissue structure by mediating cell polarity through interaction with the actin cytoskeleton [25]. Vimentin is an intermediate filament and hence a component of the cytoskeleton found in mammalian cells and expressed ubiquitously in mesenchymal cells [25]. The overexpression of Vimentin in cancers is known to correlate with accelerated tumor growth, invasion, and poor prognosis [26].

E-cadherin repression and Vimentin upregulation are regulated by the transcription factors Snail/Snai1, Slug/Snai2, Zeb1 [27]. These transcription factors were upregulated in the adherent, mesenchymal-like subpopulation of NCI-H69V cells compared to parental suspension cultures (Figures 3a,b). We observed this in the other cell lines to a lesser extent, perhaps because NCI-H82 and NCI-N592 already contain a higher proportion of adherent cells, meaning that the separation between more epithelial-like and mesenchymal-like cells was not as obvious as in the NCI-H69/NCI-H69V cells. This might also explain the expression of Vimentin in NCI-H82 suspension cells. However, Vimentin expression remains clearly increased in the adherent NCI-H82 subline. Despite small discrepancies among the three cell lines we analyzed, our overall data clearly indicate a more mesenchymal phenotype for the adherent SCLC cells. We suggest that multistep EMT processes are involved in these complex changes in phenotypes in all three cell lines, which is also supported by the epigenetic and functional changes outlined below.

Interestingly, the neuroendocrine marker expression of SCLC cells is also significantly different in adherent NCI-H69V cells compared to suspension NCI-H69 cells. Expression of NSE and L-Dopa is completely lost in adherent NCI-H69V cells (Figure 3c). We know that neuroendocrine and non-neuroendocrine subpopulations coexist in some tumors [28]. Changes in morphology are accompanied by the loss of neuroendocrine differentiation and the emergence of radio resistance [29] while Vimentin positive cells are losing their apical-basal polarity and neuroendocrine differentiation [30]. Our data concur with these earlier observations, indicating that neuroendocrine differentiation is lost during EMT of SCLC cells.

The rise in Vimentin expression has been reported to be associated with tumor migration, invasion and metastasis [31]. Polarized cell movement and matrix degradation is typical in mesenchymal migration and invasion [32]. In contrast, E-cadherin is typically expressed on epithelial cell layers that do not form metastases [33]. To form metastases, cells detach from neighboring cells, leave the tumor site, and invade blood and lymphatic vessels before settlement at the metastatic site [34]. Concurring with these descriptions of functions gained during EMT that are also linked to stemness properties [18], [35], we also observed significantly increased migratory und invasive potential in the adherent, mesenchymal-like NCI-H69V cells (Figure 4).

Furthermore, we detected a significant increase in extracellular proteolysis of collagen IV in NCI-H69V cells, whereas NCI-H69 cells growing in suspension aggregates revealed low proteolytic activity (Figure 5). This lack of proteolytic activity corresponds with their non-invasive behavior (Figure 4), as ECM degradation is a prerequisite for invasion into surrounding tissue. Matrix remodeling is mainly mediated by MMPs; a correlation between mesenchymal characteristics and higher MMP expression has been reported [36]. MMPs, especially MMP-2 and MMP-9, as well as their endogenous inhibitors (TIMPs, especially TIMP1) are known to be important in tumor development [37], [38], tumor cell invasion and metastasis. Their expression correlates with advanced clinical tumor disease stages and poor survival [39], [40]. We observed increased MMP-9, MMP-13, TIMP-1 and TIMP-2 expression and increased MMP-9 and MMP-2 activity (Figure 6). MMP-2 interacts with integrins and results in locally-enhanced matrix degradation [41] and elevated MMP-2 and MMP-9 expression is associated with tumor aggressiveness and invasion [42]. Thus we suggest that enhanced secretion and activation of distinct MMPs such as MMP-2, MMP-9 and MMP-13 are at least partially responsible for the greater invasive capacity of the adherent subpopulation.

Unlike the other MMPs, MMP-10 was decreased in adherent NCI-H69V cells compared to floating NCI-H69. In agreement with our findings, Justilien et al. reported increased expression of MMP-10 in oncospheres and a loss of MMP-10 expression in adherent cultures [43].

Besides its important role in metastasis, EMT is also known to be associated with the development of chemoresistance and early relapse in several cancer types [15]. We noted a significant increase in Etoposide resistance in the adherent, mesenchymal-like NCI-H69V cells compared to the NCI-H69 cells floating in aggregates (Figure 7). NCI-H69 and NCI-H69V cells showed comparable PDTs (Figure S1) so that chemoresistance is unlikely to be caused by a growth advantage of NCI-H69V over NCI-H69. Comparable PDTs were described before for NCI-H69 and a multidrug resistant subline, which also showed enhanced cell-cell adhesiveness [44]. Moreover, similar results on chemoresistance comparing suspension and adherent sublines were previously obtained by Kraus et al. [45] for Etoposide and to a lesser extent also for Cyclophosphamide, Doxorubicin and Paclitaxel. During treatment of patients suffering from SCLC, physicians encounter a very good initial response to chemotherapy, invariably followed by a relapse resulting in terrible five-year survival rates. Such relapses might originate from a minor SCLC subpopulation with EMT abilities. Further investigations on primary SCLC cells are needed to address the question whether the differences observed in our experiments regarding EMT-like features reflect the clinical situation.

In an effort to understand the underlying mechanism of these morphological and functional changes, we investigated the impact of epigenetic changes via DNA methylation in NCI-H69V compared to NCI-H69 cells. Epigenetic changes are involved in regulating EMT and cancer metastasis [15]. Moreover, epigenetic modifications are known to result in activating oncogenic pathways while inactivating tumor-suppressor signals [46]. There are reports that E-cadherin and Vimentin can be regulated via DNA methylation in various cancer entities [47], [48]. We therefore investigated DNA methylation levels of the Vimentin and E-cadherin promoter and found a strong inverse correlation between DNA methylation and expression in NCI-H69 and NCI-H69V cells, especially for Vimentin (Figure 3d). While other regulatory mechanisms are probably involved as well, our data demonstrate the contribution of DNA methylation in modulating the expression of key EMT-related proteins.

The investigation of SCLC is hampered by the fact that primary tumor tissue is very difficult to obtain because SCLC patients do not usually undergo surgery. In this respect, the characterization of different tumor-cell subpopulations within SCLC cell lines might at least partially reflect the *in vivo* situation with its shifting phenotypes that might recapitulate the clinical observation of high initial chemotherapy responses and refractory disease in relapse. We propose that this model deserves further examination, and that ongoing *in vivo* xenograft studies will help us address this disease's clinical and therapeutic relevance. As EMT processes seem to play a major role in metastasis and the development of drug resistance, understanding the regulating factors of EMT in SCLC is crucial to developing new therapeutic strategies.

In conclusion, we have characterized the floating and adherent subpopulations of different SCLC cell lines *in vitro* in terms of their epithelial and mesenchymal markers and their functional consequences in migration, invasion, MMP regulation and chemoresistance. This new *in vitro* cell model might prove to be a useful tool in identifying specific targets for distinct subpopulations with mesenchymal-like features such as the greater capacity to metastasize and develop drug resistance. Moreover, our study demonstrates the involvement of DNA methylation in this process, making epigenetic drugs such as the DNA-methyltransferase-inhibitors Azacytidine or Decitabine interesting candidates for new treatment options for patients with chemotherapy-resistent SCLC.

The clinical relevance of EMT and epigenetic aberrations in SCLC patients demands further investigation to clearly define new targeted therapies.

## Supporting Information

Figure S1 **Cell growth and population doubling time (PDT) of NCI-H69 and NCI-H69V.** Means of triplicates are shown. 3*10^6^ cells were seeded at day 0 and counted on day 2, 4, and 6. Similar results were obtained in several experiments.
(DOC)

Figure S2 **Analysis of MMP mRNA and protein levels in suspension cells and their adherent sublines.** Majority of analyzed MMPs, especially MMP-2, MMP-9 and MMP-14, are up-regulated in adherently growing NCI-H69V and NCI-N592adh compared to their floating counterparts NCI-H69 and NCI-N592, as shown by RT-PCR (**a**). Only MMP-10 was slightly down-regulated in NCI-H69V cells. Upregulation on protein level was confirmed by western blot for MMP-2 and MMP-14 in the NCI-H69/NCI-H69V pair (**b**).
(DOC)
