# Supplementary figures and images for: Tumor Cell Heterogeneity in Small Cell Lung Cancer (SCLC): Phenotypical and Functional Differences Associated with Epithelial-Mesenchymal Transition (EMT) and DNA Methylation Changes
Source: PLoS One. 2014 Jun 24;9(6):e100249. doi: 10.1371/journal.pone.0100249 (PMC4069054; doi:10.1371/journal.pone.0100249)

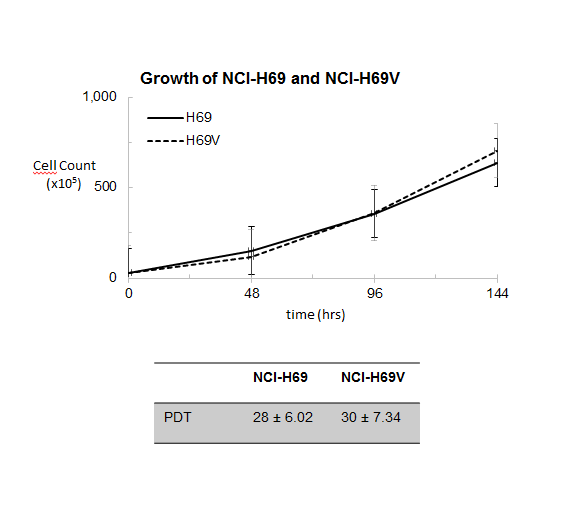


**Figure S1**

Supplement: Figure S1 — Cell growth and population doubling time (PDT) of NCI-H69 and NCI-H69V. Means of triplicates are shown. 3*106 cells were seeded at day 0 and counted on day 2, 4, and 6. Similar results were obtained in several experiments. (DOC) [file pone.0100249.s001.doc]

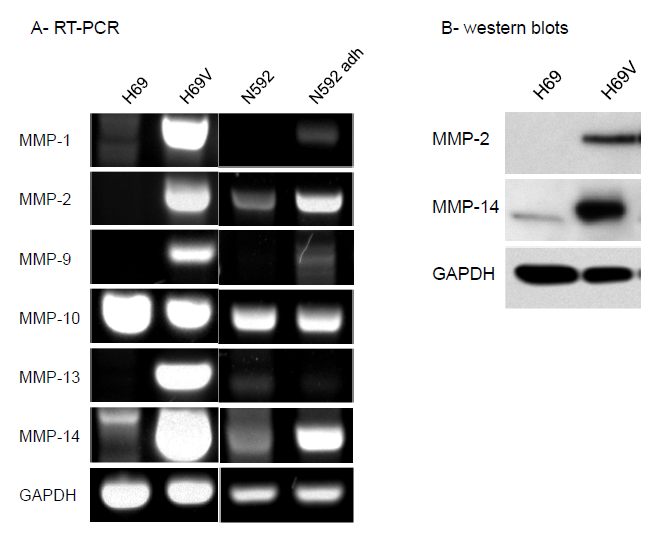


**Figure S2**

Supplement: Figure S2 — Analysis of MMP mRNA and protein levels in suspension cells and their adherent sublines. Majority of analyzed MMPs, especially MMP-2, MMP-9 and MMP-14, are up-regulated in adherently growing NCI-H69V and NCI-N592adh compared to their floating counterparts NCI-H69 and NCI-N592, as shown by RT-PCR (a). Only MMP-10 was slightly down-regulated in NCI-H69V cells. Upregulation on protein level was confirmed by western blot for MMP-2 and MMP-14 in the NCI-H69/NCI-H69V pair (b). (DOC) [file pone.0100249.s002.doc]
